# Effectiveness of a Malaria Surveillance Strategy Based on Active Case Detection during High Transmission Season in the Peruvian Amazon

**DOI:** 10.3390/ijerph15122670

**Published:** 2018-11-27

**Authors:** Diamantina Moreno-Gutierrez, Alejandro Llanos-Cuentas, José Luis Barboza, Juan Contreras-Mancilla, Dionicia Gamboa, Hugo Rodriguez, Gabriel Carrasco-Escobar, Raphaël Boreux, Marie-Pierre Hayette, Philippe Beutels, Niko Speybroeck, Angel Rosas-Aguirre

**Affiliations:** 1 Facultad de Medicina Humana, Universidad Nacional de la Amazonía Peruana, Loreto 160, Peru; hmrodriguezf@hotmail.com; 2 Research Institute of Health and Society (IRSS), Université catholique de Louvain, 1200 Brussels, Belgium; niko.speybroeck@uclouvain.be (N.S.); angelrosasa@gmail.com (A.R.-A.); 3 Centre for Health Economics Research and Modelling Infectious Diseases, Vaccine and Infectious Disease Institute, University of Antwerp, 2000 Antwerp, Belgium; philippe.beutels@uantwerpen.be; 4 Instituto de Medicina Tropical “Alexander von Humboldt”, Universidad Peruana Cayetano Heredia, Lima 31, Peru; alejandro.llanos.c@upch.pe (A.L.-C.); biobarbox@gmail.com (J.L.B.); juan.contreras.m@upch.pe (J.C.-M.); dionigamboa@yahoo.com (D.G.); 5 Facultad de Salud Pública y Administración, Universidad Peruana Cayetano Heredia, Lima 31, Peru; 6 Laboratorios de Investigación y Desarrollo, Facultad de Ciencias y Filosofia, Universidad Peruana Cayetano Heredia, Lima 31, Peru; gabriel.carrasco@upch.pe; 7 Departamento de Ciencias Celulares y Moleculares, Facultad de Ciencias y Filosofia, Universidad Peruana Cayetano Heredia, Lima 31, Peru; 8 Division of Infectious Diseases, Department of Medicine, University of California San Diego, La Jolla, CA 92093, USA; 9 Department of Clinical Microbiology, Center for Interdisciplinary Research on Medicines (CIRM), University Hospital of Liège, 4000 Liège, Belgium; raphael.boreux@chuliege.be (R.B.); mphayette@chuliege.be (M.-P.H.)

**Keywords:** malaria, diagnosis, asymptomatic, active case detection, Peru

## Abstract

*Background:* Faced with the resurgence of malaria, malaria surveillance in the Peruvian Amazon incorporated consecutive active case detection (ACD) interventions using light microscopy (LM) as reactive measure in communities with an unusual high number of cases during high transmission season (HTS). We assessed the effectiveness in malaria detection of this local ACD-based strategy. *Methods:* A cohort study was conducted in June–July 2015 in Mazan, Loreto. Four consecutive ACD interventions at intervals of 10 days were conducted in four riverine communities (Gamitanacocha, Primero de Enero, Libertad and Urco Miraño). In each intervention, all inhabitants were visited at home, and finger-prick blood samples collected for immediate diagnosis by LM and on filter paper for later analysis by quantitative real-time polymerase chain reaction (qPCR). Effectiveness was calculated by dividing the number of malaria infections detected using LM by the number of malaria infections detected by delayed qPCR. *Results*: Most community inhabitants (88.1%, 822/933) were present in at least one of the four ACD interventions. A total of 451 infections were detected by qPCR in 446 participants (54.3% of total participants); five individuals had two infections. *Plasmodium vivax* was the predominant species (79.8%), followed by *P. falciparum* (15.3%) and *P. vivax*-*P. falciparum* co-infections (4.9%). Most qPCR-positive infections were asymptomatic (255/448, 56.9%). The ACD-strategy using LM had an effectiveness of 22.8% (detection of 103 of the total qPCR-positive infections). Children aged 5–14 years, and farming as main economic activity were associated with *P. vivax* infections. *Conclusions:* Although the ACD-strategy using LM increased the opportunity of detecting and treating malaria infections during HTS, the number of detected infections was considerably lower than the real burden of infections (those detected by qPCR).

## 1. Introduction

Malaria remains an important problem in global public health with about 212 million cases and 429,000 deaths estimated worldwide in 2015 [1]. In the Americas, intensive control efforts over the past 15 years have resulted in a significant reduction in the malaria burden in the region from about 2.3 million confirmed cases and 838 malaria-related deaths in 2000 to near 800,000 cases and 490 deaths in 2015 [1]. By 2015, seven countries (Paraguay, Belize, El Salvador, Costa Rica, Mexico, Ecuador, Suriname) were attempting to eliminate malaria and many others considering doing so in the near future [1,2].

Intensified control efforts in the Amazonian Region mainly supported by the Global Fund (GF) PAMAFRO project contributed to a significant decline in malaria reported case incidence from 87,805 cases in 2005 to 23,060 cases in 2011 (about 70% reduction). Regrettably, this achievement rapidly vanished in the following years after the end of the GF project, reaching 63,153 reported cases in 2015 [3,4,5]. The vast majority of these malaria cases (about 95%) in the Peruvian territory were concentrated in Loreto Department [4]; where transmission is seasonal and highly heterogeneous, caused by *Plasmodium vivax* and *P. falciparum* (ratio of *P. vivax/P. falciparum*: 5/1) and mainly transmitted by the mosquito *Anopheles darlingi* [6,7].

Faced with the malaria resurgence in Loreto, the Peruvian government and the Regional Health Direction of Loreto (RHDL) pledged to improve the malaria case detection and case management to ensure prompt and accurate diagnosis, and opportune treatment with effective antimalarial drugs, as recommended by World Health Organization (WHO) [8].

The improved malaria action plan considered the limitations of the routine malaria surveillance relying on passive case detection (PCD) [9,10] using light microscopy (LM) for detecting both asymptomatic infected individuals (i.e., asymptomatic parasite carriers) and symptomatic individuals that do not attend the health services (i.e., symptomatic individuals with limited access to health services, and/or poor treatment-seeking behaviors) [11,12]. Indeed, several prevalence studies using LM and polymerase chain reaction (PCR) in endemic rural villages near Iquitos city (capital of Loreto) have repeatedly found that the majority (more than 60%) of the people carrying the parasite were asymptomatic. This indicates there is a large “hidden” human infectious reservoir able to maintain transmission [13,14,15].

Malaria surveillance in Loreto was enhanced by the addition of active case detection (ACD) through household visits as a reactive measure in selected communities with unusually high numbers of cases previously reported by PCD, especially during high transmission season [16]. The local strategy consists of 2–4 consecutive screenings for malaria using LM over a period of one month in all individuals living in the selected communities and the treatment of confirmed infections irrespective of symptoms. In theory, consecutive ACD interventions would increase malaria detection by microscopically identifying parasite carriers who were in a latent state at the time of the previous ACD (i.e., development of blood asexual parasites after sporozoite inoculation), and by increasing the coverage of the intervention through including individuals who were absent during the previous ACD [17]. Nevertheless, this local ACD-based strategy requires evidence on its effectiveness in different epidemiological settings before it can be implemented on a wider-scale, particularly in endemic areas where most infected individuals have low parasitemia levels below the threshold for detection by LM [18,19,20,21]. In this paper, we assessed the short-term effectiveness of this household-based malaria surveillance in four riverine communities of Mazan district in the Peruvian Amazon, in terms of the proportion of malaria infections detected by field LM in consecutive ACD interventions among the total malaria infections detected by delayed quantitative real-time polymerase chain reaction (qPCR).

## 2. Materials and Methods

### 2.1. Study Design and Study Area

A population-based cohort study was conducted in Mazan, one of the districts in Loreto with the highest annual parasite index (API): 121 cases per 1000 inhabitants in 2015 [22]. According to census projections in Mazan for 2015, 13,779 inhabitants lived in about 70 riverine communities located on the banks of Mazan or Napo River (including three communities inhabited by Orejón, Quichua and Yagua ethnic groups) [23]. People in riverine communities live mainly in open or semi-open wooden houses built on stilts. Subsistence farming is the main economic activity, followed by seasonal logging. The latter activity requires individuals to stay far out in the jungle for several weeks during the rainy season, where they usually sleep unprotected against mosquito bites.

The capital and largest community of Mazan, i.e., Mazan Town (MT), is located on the confluence between Mazan and Napo Rivers (3.503° S, 73.094° W), at about 55–60 km (one hour by speedboat) from Iquitos city (capital of Loreto). Due to its geographic situation, MT mediates the commerce between all communities located in margin of the Napo River and Iquitos city. With predominance of *P. vivax* (76.2%) over *P. falciparum* cases (23.8%), malaria in Mazan is seasonal, epidemic [24], and mainly transmitted by the highly anthropophilic *An. darlingi* [25]. All age groups are at risk of infection, though adults more so than children. The peak of cases is usually after the rainy season [6], between May and September. The main Health Centre in Mazan is also located in MT, and has a laboratory service responsible for providing malaria diagnosis using LM and complete treatment in confirmed malaria infections six days a week. Malaria diagnosis and treatment in the area follow MoH standard procedures and treatment guidelines. Chloroquine (CQ) for 3 days (10 mg/kg on days 1 and 2, and 5 mg/kg on day 3 plus primaquine (PQ) (0.5 mg/kg/day) for 7 days is given in individuals with confirmed *P. vivax* malaria infections, while mefloquine (MQ) for 2 days (12.5 mg/kg/day) plus artesunate (AS) for 3 days (4 mg/kg/day) in confirmed *P. falciparum* infections [11]. A number of drug trials have confirmed the efficacy of CQ for *P. vivax* and of the combination MQ-AS for *P. falciparum* in the Peruvian Amazon [26,27].

### 2.2. Selection of Communities

Ten communities that accounted for about 80% of the total malaria cases in the district in the past two years (2013–2014) were censused and their eight-week slide positivity rates (8w-SPR) weekly analysed since the first week of March 2015. The 8w-SPR was weekly calculated by dividing the number of microscopically confirmed slides/cases in the past 8 weeks according to PCD surveillance by the total individuals screened for malaria by thick/thin blood slides in the past 8 weeks.

The evolution of 8w-SPR values in malaria endemic communities is considered in the decision of RHDL for implementing additional control interventions [9,10,28]; thus, 8w-SPR values above the threshold of 0.05 in two consecutive weeks indicating unusual increase of malaria cases (potential outbreak) commonly trigger the onset of ACD interventions. 

**Figure 1:**
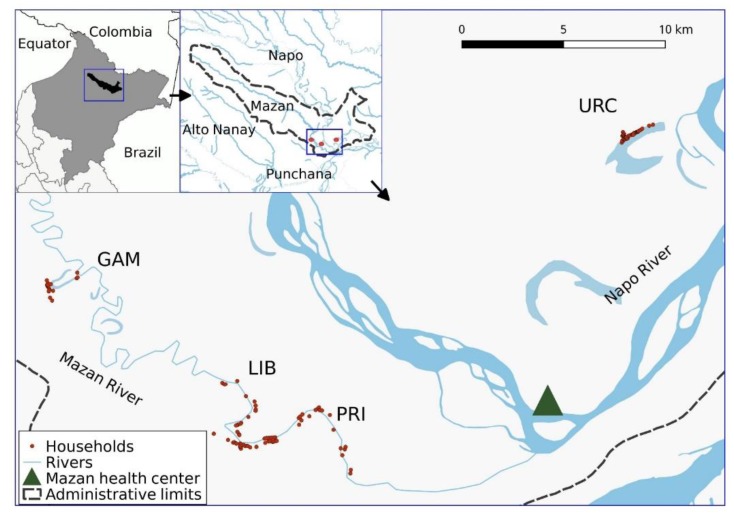
Study communities in Mazan, Loreto, Peru. Gamitanacocha (GAM), Primero de Enero (PRI), Libertad (LIB) and Urco Miraño (URC).

By May 2015, four communities exceeded the triggering threshold and were selected for the cohort study: Gamitanacocha (GAM), Primero de Enero (PRI), Libertad (LIB) and Urco Miraño (URC). GAM (3.426°S, 73.318°W), PRI (3.494° S, 73.221° W) and LIB (3.496° S, 73.234° W) are located along Mazan River, while URC (3.361° S, 73.064° W) along Napo River. They are only reachable from MT (about 3–5 h upstream) using a 9–13 hp motor on a dugout canoe, locally known as a “peke-peke” (Figure 1). Only LIB has a health post, where health technicians can take a finger-prick of blood from individuals with malaria-like symptoms and subsequently send the blood smears to the main health centre in MT for malaria diagnosis (no laboratory service in LIB). The health post in LIB can also provide antimalarial treatment to individuals with confirmed infections referred from the main heath centre in MT.

### 2.3. ACD Interventions and Data Collection

During the full population census in March 2015, each household in the selected communities was encoded (6-digit numeric code) and geo-referenced using a Global Positioning System (GPS) hand device (Garmin’s GPSMAP 60CSx, Garmin International Inc., Olathe, KS, USA).

In June–July 2015, four consecutive ACD interventions were conducted at intervals of 10 days in selected communities. In each ACD intervention, all community inhabitants were visited at home, and after informed consent they had axillary temperature taken, history of fever or any other malaria-compatible symptoms documented through use of a semi-structured questionnaire, and finger-prick blood samples collected for immediate microscopy (thick and thin blood smears) and on filter paper (Whatman grade 3, Whatman, Springfield Mill, Philadelphia, PA, USA) for later analysis by qPCR (two–three months after the field-work). Household visits also allowed the collection of household and individual data (e.g., household characteristics, age, education, gender, socioeconomics, recreation and occupational activities, malaria history, previous antimalarial treatment, bed net use, travel) through questionnaires programmed onto Open Data Kit (ODK) application on mobile devices without network connection. Data were synchronized with the project’s server in Lima, and linked to laboratory results using 9-digit unique numerical codes.

ACD interventions were designed and coordinated with health authorities and workers from RHDL and the health centre in MT. Field teams included interviewers and nurses in charge of the data entry and sample collection, microscopists and laboratory technicians dealing with the diagnostic tasks, and trained health technicians from the health centre in MT who were in charge of the malaria case management including the treatment of all microscopically confirmed infections according to national guidelines. Between ACD interventions, community health volunteers visited individuals with microscopically confirmed infections to improve the adherence to the antimalarial treatment. Field teams travelled to the communities in motorized boats and settled there for a few days (staying overnight in semi-closed huts and/or tents). During the screenings, the field staff moved around with the boats to reach the scattered households (most of them surrounded by water) and, by the time samples were sequentially delivered at the community “base-camp”, where microscopists proceeded with the blood-slide diagnostic by using mirror-illuminated microscopes (no electricity available). Households were visited up to three times in a period of 3 days to maximize subject participation in each ACD intervention.

### 2.4. Laboratory Procedures

#### 2.4.1. Light Microscopy (LM)

Microscopy examination was performed the same day of sample collection in each ACD survey. Thick and thin smears were stained for 10 min with a 10% Giemsa solution. A slide was judged to be negative if no malaria parasite was seen after examining 100 high-powered fields [29]. Both the presence of asexual and sexual forms of *Plasmodium* species were determined during slide readings. Quality control was done blindly on all positive slides and 10% of randomly chosen negative slides by a senior technician at Reference Laboratory of RHDL. Parasite density in the field was reported using semi quantitation system (“plus system” scale) according to MoH standard procedures and national guidelines [29]: <+/2 (<40 parasites per 100 thick films fields); +/2 (40 to 60 parasites per 100 thick films fields); + (1 parasite per one thick film field); ++ (2 to 20 parasites per one thick film field); +++ (21 to 200 parasites per one thick film field); ++++ (>200 parasites per one thick film field).

#### 2.4.2. Quantitative Real-Time Polymerase Chain Reaction (qPCR)

Filter paper areas containing blood were cut into ~6 mm^2^ sections for the parasite genomic DNA extraction using E.Z.N.A.^®^ Blood DNA Kit (Omega Bio-Tek^®^, Norcross, GA, USA), following manufacturer instructions with slight modifications—addition of TEN (20 mM Tris-HCl, pH 8.0; 2 mM EDTA, pH 8.0; 0.2 M NaCl) buffer, supplemented with SDS 10% w/v—and stored at 4 °C for immediate use and at −20 °C for further analysis.

qPCR testing was done following a modified protocol from Mangold et al. [30] using PerfeCta SYBR Green Fast Mix (Quanta Biosciences, Gaithersburg, MD, USA). Briefly, this method amplifies the 18SSU rRNA gene sequence of the *Plasmodium* species-specific region. After the amplification, analysis of the differences in melting curves provided an accurate differentiation between *Plasmodium* species.

#### 2.4.3. Multiplex Real Time PCR (mPCR)

For improving the detection of *P. vivax/P. falciparum* co-infections, all samples of individuals who presented consecutive positive qPCR results in which the identification of *Plasmodium* species changed across ACD visits—in absence of previous antimalarial treatment—were further analysed using the mPCR assay described by Cnops et al. [31]. Briefly, DNA was extracted from dry blood spots using Qiagen DNA mini kit (Qiagen Benelux, Venlo, The Netherlands), according to the manufacturer’s guidelines except the addition of 10 µL of an internal control (DiaControlDNA, Diagenode, Liège, Belgium). A couple of primers were used to detect *P. vivax* (Pviv) and *P. falciparum* (Pfal) in combination with the 2 species-specific probes and reverse plasmo2 primer (Eurogentec, Seraing, Belgium). Primers and probes of DiacontrolDNA were used to test the extraction and amplification process. The mix consists of 12.5 µL of 2x Taqman Universal PCR master mix (Applied Biosystems, Foster City, CA, USA), 200 nM of Pfal and *P. falciparum* specific probe, 100 nM of Pviv and *P. vivax* specific probe, 2.5 µL of DiaControlDNA primers and probes and 5 µL of DNA extract. Water was added to obtain a final volume of 25 µL. PCR conditions were as follows: initial step of 50 °C for 2 min, 95 °C for 10 min and 45 cycles of 95 °C for 15 s followed by 1 min. at 60 °C. PCR was run on an ABI 7500 thermocycler (Applied Biosystems, Foster city, CA, USA).

### 2.5. Data analysis

The analysis was performed using STATA 12.1 software (Statacorp, College Station, TX, USA) and R v.2.15 software (R Development Core Team, R Foundation for Statistical Computing, Vienna, Austria). Baseline characteristics between communities were compared using the Chi-squared test.

All four ACD interventions/visits were taken into account to characterize malaria infections in study participants. A positive qPCR result (regardless of the presence of symptoms) defined a malaria infection in a participant, and the number of ACD visit (1st, 2nd, 3rd or 4th) in which the qPCR was positive considered as the time of detection. A consecutive positive qPCR result in the same participant at the following ACD visit (after 10 days) was considered as still being part of the infection detected at the previous ACD visit, even when the *Plasmodium* species detected on consecutive visits were different (*P. vivax* or *P. falciparum*). In this latter situation, in absence of antecedent of antimalarial treatment, the infection was considered as produced by co-infection *P. vivax*-*P. falciparum*. Two new independent infections in a participant were only considered when the participant had an earliest ACD visit with both positive qPCR and LM results (with proven administration of antimalarial treatment), and a later non-consecutive ACD visit (after 20 or 30 days) with positive qPCR result regardless of the parasite species. An infection was classified as symptomatic if the individual had axillary temperature higher than 37.5 °C at the time of its detection by qPCR, reported history of fever in the previous seven days, or had headache, chills or general discomfort in the previous 24 h [11,32].

The effectiveness in malaria detection of the local ACD-based strategy was calculated by dividing the number of malaria infections detected through consecutive ACD interventions using LM by the total number of malaria infections detected by qPCR throughout the study period. When the local ACD-based strategy was able to detect individuals who had malaria infections, the time of this detection by LM (1st, 2nd, 3rd or 4th visit) was registered and later compared with the time of detection using qPCR. Uni- and multivariate mixed-effects logistic regression models were used to determine risk factors for species-specific malaria infection. The following potential risk factors were assessed as fixed-effects in the models: age, gender, occupation, travel in the past month to another endemic area, the history of malaria in the past year, housing structure (number of wall), electricity availability and indoor residual insecticide spraying in the past year. Random effects were included in the models to account for individuals nested within households, and households nested within communities. Factors with *p* values < 0.1 for the Wald test in the univariate analysis were considered for inclusion in the full multivariate model. Interactions were systematically checked for up to order two. Manual backward elimination guided by the minimization of the Akaike information criterion (AIC) allowed for the reduction of the number of factors in final models. Likelihood ratio tests (LRTs) were used to assess statistical differences between nested models.

### 2.6. Ethical Issues

Ethics clearance for the study was obtained from the Ethics Review Board of the Universidad Peruana Cayetano Heredia, Lima, Peru (SIDISI code # 64371). Permissions were received from health and local authorities after explaining the purpose and procedures of the study. Signed informed consent was obtained prior the study enrollment to participation and blood sampling by all adults and the parents of all participating children <18 years. In addition to parental/guardian consent, children older than 7 years provided a signed informed assent. All the methods were carried out in accordance with approved guidelines.

## 3. Results

### 3.1. Participants

Among the total 933 censused people—living in 158 households—in March 2015 in the study area, 822 (88.1%) people—living in 154 households—were surveyed and sampled during at least one of the four consecutive ACD visits conducted in June–July 2015, with the following distribution by community: GAM (92 individuals, 91.1% of censused people), LIB (299, 86.2%), PRI (100, 82.6%) and URC (331, 90.9%) (Table 1). While intervention coverages exceeded 70% in individual ACD visits (range between 73.4% and 78.5%), censused people that completed the four ACD visits only reached 58.3% with the lowest figures in GAM (43.6%) and PRI (44.6%).

Baseline socio-demographic characteristics and history of past malaria episodes of participants are presented in Table 2.

Males slightly outnumbered females (ratio female/male: 0.91) without differences between sites. Individuals < 15 years represented 47.2% of the total participants with slight differences in the proportion of this age group across communities (*p* = 0.117). Among adults 18 years and older, the proportion of individuals who had completed secondary/higher education level was higher in URC (33.8%) than in other communities (*p* = 0.014). The most commonly reported occupation among individuals 15 years and older was farming (70.1%), with a greater proportion of this economic activity in GAM and PRIM (*p* = 0.044). At the time of the first ACD visit, participants reporting having experienced malaria in the past 12 months were more common in GAM (28.6%), LIB (23.2%) and URC (21.8%) than in PRIM (20.0%) (*p* = 0.495). Bed net use the previous night was almost universal in all sites, and about 10% of people reported that they had travelled outside the community in the previous 4 weeks. The proportion of individuals having electricity (81.3%) and reporting indoor residual spraying in the past 12 months at home (99.7%) were higher in URC than in other communities (*p* < 0.001), while housing structure with 4 walls was less frequent in PRI (5.0%) than in other communities (*p* < 0.001).

### 3.2. Malaria Infections in the Study Period

Overall, 451 malaria infections were detected by qPCR in 446 participants (54.3% of total participants) through the four ACD visits (Table 3): 40.4% (182/451) infections were detected at the first visit, 22.4% (101/451) at the second visit, 20.6% (93/451) at the third visit, and 16.6% (75/451) at the fourth visit. About 80% (360/451) of these infections were due to *P. vivax*, 15.3% (69/451) due to *P. falciparum*, and 4.9% (22/451) considered as coinfections *P. vivax*-*P. falciparum*. The latter coinfections were detected in 22 individuals who had change in the species detection by qPCR in consecutive ACD visits. Additional analysis of these individuals’ samples using mPCR detected DNA of both species in two individuals at a same visit (mixed infections). According to the case definitions, five participants were considered as presenting two malaria infections during the study period: all had recurrent *P. vivax* infections between 20 and 30 days after presenting a primary *P. vivax* infection treated by health workers with standard antimalarial regimen (CQ plus PQ).

**Figure 2:**
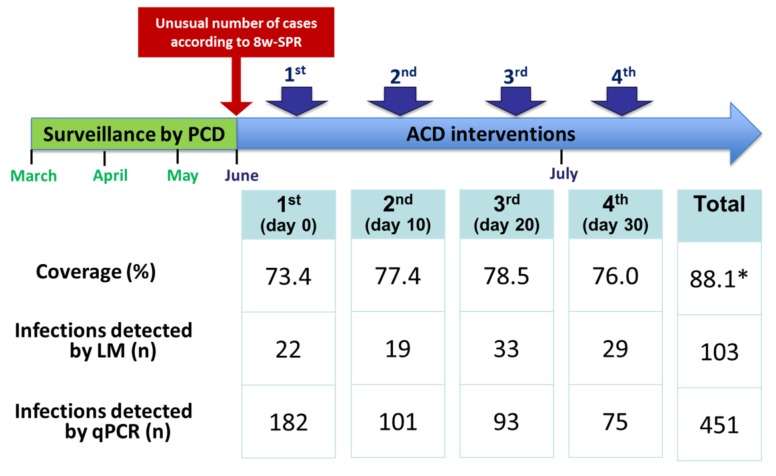
Effectiveness in malaria detection of the ACD local strategy. * Proportion of individuals with at least one ACD visit; eight-week slide positivity rate (8w-SPR); passive case detection (PCD); active case detection (ACD); light microscopy (LM); quantitative real-time polymerase chain reaction (qPCR).

Table 3 relates the time of detection of malaria infections by qPCR with the time of field detection by LM during the four-ACD local strategy and Figure 2 presents the effectiveness in malaria detection of the ACD local strategy. Only 103 (22.8%) of the total 451 qPCR-positive malaria infections were accurately detected by LM despite the intensive case detection efforts (i.e., four consecutive ACD visits using LM): 67 (65.0%) at the same ACD visit and 36 (35.0%) at the following ACD visits.

Among these 103 infections detected by LM, 99 were *P. vivax* infections (27.5% of the total *P. vivax* infections detected by qPCR), three were *P. falciparum* infections (4.3% of the total *P. falciparum* infections detected by qPCR), and one *P. vivax*-*P. falciparum* coinfection (4.5% of the total co-infections detected by qPCR). The majority of individuals with microscopically-confirmed infections (>90%) reported had completed the treatment and had been followed by community health workers. Interestingly, the number of infections detected by qPCR in the first ACD visit (182 infections) was considerably higher than that detected by the four-ACD local strategy using LM (103 infections). The specific tracking of LM results from the 182 individuals who presented qPCR-positive infections at the first ACD visit found that only 22 (12.1%) individuals were accurately detected by LM at the same first ACD visit: 20 had *P. vivax* infections and 2 *P. falciparum* infections. Another 22 (12.1%) individuals with qPCR-positive infection at the first ACD visit were also detected by the local ACD-based strategy but in the following visits: 9 individuals had confirmed malaria infection by LM at the second visit (10 days later), 5 individuals at the third visit (20 days later), and 8 individuals at the fourth visit (30 days later) (Table 3).

The analysis of available parasite density data of detected infections by LM showed that most microscopically confirmed *P. vivax* infections (59.8%, 58/97) had low parasite density levels (<1+ in the “plus system” scale) and that all three microscopically confirmed *P. falciparum* infections had parasitaemia ≥1+ (i.e., ≥1 parasite per one thick film field). The unique coinfection accurately detected by LM had very low parasite densities (<1/2+). Four additional individuals that had *P. vivax*-*P. falciparum* co-infections were inaccurately detected by LM as *P. vivax* mono-infections (Table 4).

Table 5 presents the evolution of clinical status of malaria infections detected by qPCR, overall and by species. Only 128 (28.6%) of the total 448 PCR-positive malaria infections were symptomatic at the time of their detection by qPCR: 108 were *P. vivax* infections (30.2% of the total 358 *P. vivax* infections with clinical data), 16 *P. falciparum* infections (23.5% of the total 68 *P. falciparum* infections with clinical data), and four *P. vivax-P. falciparum* coinfections (18.2% of the total 22 co-infections). Incomplete data about symptoms in three malaria infections (two *P. vivax* infections and one *P. falciparum* infection) did not allow for the definition of their clinical status.

Of the total 179 individuals with positive qPCR results at the first visit, 76 (42.5%) had symptoms at the same visit and another 30 (16.8%) individuals developed symptoms at later visits: 18 (10.1%) at the second, 10 (5.6%) at the third and 2 (1.1%) at the fourth visit. Noteworthy, an important proportion of individuals with qPCR-positive infections (40.8%, 73 individuals) at the first visit remained asymptomatic along the four ACD visits (Table 5). This latter proportion varied slightly in individuals with infections detected by the local ACD-based strategy (35.7%) and in those non-detected by the local ACD-based strategy (42.3%) (*p* = 0.47) (Supplementary Materials: Table S1).

### 3.3. Risk Factors by Malaria Species

Uni- and multivariate mixed effects logistic models for species-specific malaria incidence are presented in Table 6. In the final models, the estimates of the random-effects coefficients confirmed the influence of clustered sampled data at the community level for *P. vivax* infection, and at the household level for *P. falciparum*. Age and agricultural economic activities remained independently associated with *P. vivax* malaria infection in the multivariate model. While individuals between 5 and 14 years (AOR 1.7, 95% CI [1.1–2.6]) had higher odds of *P. vivax* infection than those <5 years, farmers were 1.6 times more likely to have a *P. vivax* infection than participants involved in other economic activities (AOR 1.6, 95% CI [1.0–2.4]). Considering *P. falciparum*, a non-significant trend was found for males and older adults to have higher risk of infection than women and young children, likely due to the low number of *P. falciparum* infections.

## 4. Discussion

This short-term population-based cohort study showed that the local ACD-based strategy in riverine Amazonian communities was able to detect about five times more microscopically-confirmed malaria infections during a high transmission season (HTS) than an intervention based on a single ACD visit. Nevertheless, detected microscopic malaria infections by this strategy only represented 22.8% of the total malaria infections detected by a delayed qPCR analysis of collected blood samples during ACD visits. Indeed, the latter highly sensitive test confirmed that more than half of the participants acquired mono-infections due to endemic *Plasmodium* parasites (*P. vivax*/*P. falciparum* ratio: 5.2/1) at any of the ACD visits, but also evidenced that co-infections by both species would be more common than previously reported in the Peruvian Amazon. Moreover, the clinical follow-up found that most qPCR-positive infections were asymptomatic and sub-microscopic at the time of their detection, and that only a small proportion of them became symptomatic and detectable by LM in following ACD visits.

Four consecutive ACD visits at intervals of 10 days increased the total screened population by LM from 73.4% (on the first ACD visit) to 88.1% (on any of the four ACD visits). This meant that most individuals had two or more screenings (83.3% of censused people, 94.6% of people with at least one ACD visit) during the intervention period. With this 30-day extended coverage, the local ACD-based strategy detected significantly more individuals with microscopic malaria infections than an intervention based on a single ACD visit (first visit), included: absent individuals on the first visit (mainly due to travel and economic activities) whose malaria infection was detected by LM at later visits, individuals with latent infections at the first visit who developed microscopically detectable asexual parasites in blood at later visits, individuals with submicroscopic infections at the first visit that had higher parasite density levels detectable by LM on later visits, and individuals that acquired microscopic infections after the first visit [18,25]. The latter case implies that latent periods for both *P. falciparum* and *P. vivax* (i.e., between sporozoite inoculation and development of asexual blood stage) are shorter than the time gap between the first and the fourth ACD visit [33].

The predominance of low-density infections among the total microscopically-confirmed malaria infections anticipated some limitation in the performance of the local ACD-based strategy. The availability of qPCR results at each ACD visit not only confirmed that the local strategy missed 77.2% of the total qPCR-positive infections detected throughout the study period, but also that a single ACD intervention using qPCR would be able to detect about 40% more infections in study participants than four consecutive ACD interventions using LM. Previous studies in the Peruvian Amazon had consistently found that the large majority of prevalent malaria infections by both *P. vivax* and *P. falciparum* could only be detected by highly sensitive diagnostic tests, but their cross-sectional designs did not allow the estimation of the proportion of infections that later exceed the parasite threshold for LM detection [13,14,34]. In our study, the follow-up through consecutive ACD visits found that only a small proportion of sub-patent infections—with positive qPCR and negative LM results—at the first ACD visit (13.8%, 22/160) became patent and detectable by LM in subsequent visits, suggesting a persistence of sub-microscopic parasite carriers.

This persistence has recently been pointed out as an important contributor for onward transmission in the Amazon region, since it likely harbors a “hidden” human infectious reservoir that remains largely undetected by conventional malaria surveillance, and therefore untreated [35,36]. Indeed, several reports using mosquito feeding-assays have indicated that mosquitoes can be infected with low-density *P. falciparum* and *P. vivax* infections, although less efficiently than with high-density infections [37]. Although conclusive data on the natural history of low-density malaria infections in different endemic settings remain elusive [38], mathematical modelling could be used to understand how the burden and persistence over time of sub-microscopic infections can compensate the lower efficiency of these infections in infecting mosquitoes [39]. Given the unknown measurable parasite density threshold below which transmission cannot occur, WHO recommends in low-transmission and elimination settings that all malaria infections (including those infections with low-density parasitaemia) should be considered as potentially infectious [38].

Data regarding the clinical evolution of malaria infections also showed that some individuals with qPCR-positive infections who were asymptomatic on the first ACD visit became symptomatic over the subsequent 30 days of follow-up, reducing the proportion of asymptomatic infections from about 60% (when only clinical data of the first visit is considered) to about 40% (when clinical data of all ACD visits is considered). Few longitudinal studies in Amazonia have reported data of this clinical evolution. For instance, a 6-months cohort study in Zungarococha village in the Peruvian Amazon found a high proportion of malaria infections which were asymptomatic at the time of detection but gave rise to malaria symptoms after one week of follow-up, resulting in about 40% of asymptomatic infections among all confirmed cases in study communities [13]. Another study conducted in Northwestern Brazil in 2004–2005 [40], combining PCD and ACD methods over a 14-month period, found that among 93 asymptomatic qPCR-positive infections during surveys, only 10 (10.7%) developed symptoms over the subsequent 2 months of follow-up. More evidence about the clinical evolution of sub-patent infections will be provided soon by population-based longitudinal studies implemented in the Amazonia with the aim of assessing to what extent persistent asymptomatic and sub-patent infections contribute to malaria transmission [35,36].

As in the routine malaria surveillance, the *P. vivax*/*P. falciparum* ratio (5.2/1) in our study confirmed *P. vivax* as the predominant species in Mazan district. In co-endemic areas for both species like Vietnam [41], Brazil [42] and Peru [14], *P. vivax* infections tend to have lower parasitaemia and less symptoms than *P. falciparum* infections as result of different species-specific patterns in the acquisition of host immunity (i.e., earlier and faster development of malaria immunity for *P. vivax* than for *P. falciparum*) [43]. It is not clear how hypnozoite-triggered relapses representing most *P. vivax* infections/recurrences according to modelling estimations [44,45] can influence the development of malaria immunity, but treatment-time-to-infection studies have reported that most *P. vivax* recurrences were submicroscopic and asymptomatic [43,46,47].

Moreover, lower parasite densities in *P. vivax* infections can also be explained by the inability of *P. vivax* to infect all age classes of erythrocytes in comparison with *P. falciparum* [48]. The lower proportion of asymptomatic and submicroscopic infections among the total qPCR-positive *P. vivax* infections (59.8% and 72.5%) in comparison with that among qPCR-positive *P. falciparum* infections (69.2% and 95.7%, respectively) found in our study reflects the differences in transmission intensity by species in the selected communities during the study period. Indeed, the main criterion for the selection of study communities was the confirmation of a recent and unusual increase of reported malaria cases in communities (which was mainly due to *P. vivax*) with the historically highest malaria risk in Mazan, and this confirmation immediately triggered the onset of ACD interventions. Therefore, it is probable that the study period captured the onset of malaria outbreaks in the communities, caused predominantly by *P. vivax*. Indeed, findings from the risk factor analysis showing the highest risk for *P. vivax* infection in individuals with increased exposure to infective vector bites following outdoor- and mobility-related occupational activities [49] (i.e., adult farmers and children who usually start their participation in these activities as early as at 5–7 years [15,25]), suggested that an important proportion of the total detected *P. vivax* infections in our study might be newly acquired infections rather than hypnozoite-triggered relapses. A different study design such as a treatment-time-to-infection trial incorporating molecular genotyping methods may help to estimate the contribution of relapses/new infections to the total *P. vivax* infections [50,51], however this was not the case in our study. Occupational mobility patterns could also have facilitated the introduction and spread of new parasite strains across communities [52], leading to an increase of symptomatic and microscopically detectable malaria infections in non-immune individuals when conditions for transmission were favourable [17].

Given the limitations of molecular tests in the detection of very low parasitaemia using blood spot filter papers [53], all qPCR results in ACD visits were taken into account to characterize malaria infections in the participants. In this regard, the detection of mixed/co-infections posed a major challenge since molecular tests like qPCR frequently identify only one species (the species with the most abundant parasite DNA) [54]. Therefore, any change in the species detection by qPCR in consecutive ACD visits in study participants without history of antimalarial treatment (22 participants representing 4.9% of the total qPCR-positive infections) was considered as *P. falciparum*/*P. vivax* co-infection. This operative definition could overestimate the proportion of *P. falciparum*/*P. vivax* co-infections. However, DNA detection of both species in samples of two participants in a same visit (mixed infections) by a highly specific mPCR [31] suggests that *P. falciparum*/*P. vivax* co-infections in some communities of the Peruvian Amazonian are more common than previously reported in the routine malaria surveillance and observational studies [14,15]. Indeed, cross-sectional surveys in endemic communities around Iquitos have found that less than 1% of total infections detected by PCR were mixed infections missed by LM [14], and more recently a study that used PCR to analyse packed red blood cell (PRBC) samples of 450 individuals during a malaria outbreak in two Amazonian communities did not identify any mixed infection [55].

## 5. Conclusions

In conclusion, although the local ACD strategy based on repeated and consecutive population screenings using LM increased the opportunity of detecting and treating microscopic and symptomatic malaria infections in selected Amazonian communities during HTS, our study showed that the number of detected infections was considerably lower than the number of detected infections by qPCR consisting mainly of sub-microscopic and asymptomatic infections.

According to our study, a single one ACD using qPCR would be more effective in detecting malaria infections than the four-ACD strategy using LM. This finding should encourage Peruvian malaria stakeholders to assess the feasibility and cost-effectiveness of the incorporation of molecular tests in the malaria case detection activities for reducing malaria transmission and burden (considering different operational criteria for the implementation and malaria transmission scenarios).

These assessments may include non-conventional molecular tests based on loop-mediated isothermal amplification (LAMP) which has recently proven to provide accurate detection of asymptomatic malaria parasite carriers in isolated Amazonian communities that lack of laboratory infrastructure. Alternative interventions like mass drug administration (MDA) using effective treatment combinations for both species could also be evaluated, especially in scenarios where more than half the population acquired the infection over a short time period (outbreaks, peaks of the high transmission season). Mathematical modelling can be used to assess these tools and strategies in terms of geographic scope and long-term feasibility in different malaria endemic settings.

## Figures and Tables

**Table 1:** Number of visits and participants in census by community.

		Community				
		GAM	LIB	PRI	URC	Total
**Households**						
In census	*N*	21	58	20	59	158
With at least one ACD visit	*n* (%)	19 (90.5)	56 (96.6)	20 (100)	59 (100)	154 (97.5)
**Individuals**						
In census	*N*	101	347	121	364	933
With at least one ACD visit	*n* (%)	92 (91.1)	299 (86.2)	100 (82.6)	331 (90.9)	822 (88.1)
With one visit	*n* (%)	11 (10.9)	25 (7.2)	4 (3.3)	4 (1.1)	44 (4.7)
With two visits	*n* (%)	25 (24.8)	24 (6.9)	10 (8.3)	15 (4.1)	74 (7.9)
With three visits	*n* (%)	12 (11.9)	63 (18.2)	32 (26.4)	53 (14.6)	160 (17.1)
With four visits	*n* (%)	44 (43.6)	187 (53.9)	54 (44.6)	259 (71.2)	544 (58.3)
In the 1st visit	*n* (%)	64 (63.4)	242 (69.7)	67 (55.4)	312 (85.7)	685 (73.4)
In the 2nd visit	*n* (%)	63 (62.4)	255 (73.5)	95 (78.5)	309 (84.9)	722 (77.4)
In the 3rd visit	*n* (%)	73 (72.3)	258 (74.4)	87 (71.9)	314 (86.3)	732 (78.5)
In the 4th visit	*n* (%)	73 (72.3)	255 (73.5)	87 (71.9)	294 (80.8)	709 (76.0)

ACD: active case detection; GAM: Gamitanacocha; PRI: Primero de Enero; LIB: Libertad; URC: Urco Miraño.

**Table 2:** Baseline socio-demographic characteristics of study participants by community.

Characteristics		Gamitanacocha (GAM)		Libertad (LIB)		Primero de Enero (PRI)		Urco Miraño (URC)		Total	
		*N* = 92		*N* = 299		*N* = 100		*N* = 331		*N* = 822	
		*n*	%	*n*	%	*n*	%	*n*	%	*n*	%
Gender											
	Male	43	46.7	159	53.2	55	55.0	174	52.6	431	52.4
Age (years)											
	<5	18	19.6	56	18.7	16	16.0	56	16.9	146	17.8
	5–14	23	25.0	72	24.1	34	34.0	113	34.1	242	29.4
	15–39	32	34.8	101	33.8	32	32.0	82	24.8	247	30.1
	≥40	19	20.7	70	23.4	18	18.0	80	24.2	187	22.8
Education (≥18 years) * *n* = 391											
	None	3	6.7	24	15.8	5	10.6	20	14.7	52	13.7
	Primary school	33	73.3	102	67.1	33	70.2	70	51.5	238	62.6
	Secondary school or higher	9	20.0	26	17.1	9	19.2	46	33.8	90	23.7
	Missing			8				3		11	
Main occupation (≥15 years) *											
	None or others	10	19.6	49	28.7	11	22.0	60	37.0	130	30.0
	Farmer	41	80.4	122	71.4	39	78.0	102	63.0	304	70.1
Malaria episodes (previous 12 months)											
	0	65	71.4	229	76.9	80	80.0	259	78.3	633	77.2
	≥1	26	28.6	69	23.2	20	20.0	72	21.8	187	22.8
	Missing	1		1						2	
Travel (previous month) *											
	No	80	87.0	269	90.0	81	81.0	306	92.5	736	89.5
	Yes	12	13.0	30	10.0	19	19.0	25	7.6	86	10.5
Housing structure (number of walls) *											
	0	10	11.0	42	14.2	16	16.0	44	13.3	112	13.7
	1–3	53	58.2	207	70.2	79	79.0	188	56.8	527	64.5
	4	28	30.8	46	15.6	5	5.0	99	29.9	178	21.8
	Missing	1		4						5	
Indoor residual spraying (previous 12 months) *											
	No	14	15.4	28	9.49	10	10.0	1	0.31	53	6.5
	Yes	77	84.6	267	90.5	90	90.0	325	99.7	759	93.5
	Missing	1		4				5		10	
Electricity available *											
	No	67	73.6	215	72.9	69	69.0	62	18.7	413	50.6
	Yes	24	26.4	80	27.1	31	31.0	269	81.3	404	49.5
	Missing	1		4						5	

* *p* < 0.05.

**Table 3:** Time of detection of malaria infections by quantitative real-time polymerase chain reaction (qPCR) and light microscopy (LM).

Time of Detection of Malaria Infections by qPCR		Time of Detection of Malaria Infections by the Local Strategy Using LM					
		Same Visit	Next Visit (10-days Later)	After 2 Visits (20-days Later)	After 3 Visits (30-days Later)	Non Detected	Total
		*n* (%)	*n* (%)	*n* (%)	*n* (%)	*n* (%)	*N*
Overall							
	1st visit	22 (12.1)	9 (4.9)	5 (2.7)	8 (4.4)	138 (75.8)	182
	2nd visit	10 (9.9)	6 (5.9)	4 (4.0)	ND	81 (80.2)	101
	3rd visit	22 (23.7)	4 (4.3)	ND	ND	67 (72.0)	93
	4th visit	13 (17.3)	ND	ND	ND	62 (82.7)	75
	Total	67 (14.9)	19 (4.2)	9 (2.0)	8 (1.8)	348 (77.2)	451
*P. vivax*							
	1st visit	20 (14.5)	9 (6.5)	4 (2.9)	7 (5.1)	98 (71.0)	138
	2nd visit	10 (12.8)	6 (7.7)	4 (5.1)	ND	58 (74.4)	78
	3rd visit	22 (26.5)	4 (4.8)	ND	ND	57 (68.7)	83
	4th visit	13 (21.3)	ND	ND	ND	48 (78.7)	61
	Total	65 (18.1)	19 (5.3)	8 (2.2)	7 (1.9)	261 (72.5)	360
*P. falciparum*							
	1st visit	2 (6.7)	0 (0)	1 (3.3)	0 (0)	27 (90.0)	30
	2nd visit	0 (0)	0 (0)	0 (0)	ND	17 (100.0)	17
	3rd visit	0 (0)	0 (0)	ND	ND	8 (100.0)	8
	4th visit	0 (0)	ND	ND	ND	14 (100.0)	14
	Total	2 (2.9)	0 (0)	1 (1.4)	0 (0)	66 (95.7)	69
Co-infection *							
	1st visit	0 (0)	0 (0)	0 (0)	1 (7.1)	13 (92.9)	14
	2nd visit	0 (0)	0 (0)	0 (0)	ND	6 (100.0)	6
	3rd visit	0 (0)	0 (0)	ND	ND	2 (100.0)	2
	4th visit	0 (0)	ND	ND	ND	0 (0)	0
	Total	0 (0)	0 (0)	0 (0)	1 (4.5)	21 (95.5)	22

* P. vivax-P. falciparum. ND: no data.

**Table 4:** Parasite density level at the time of detection malaria infection.

Parasite Density Level (Plus System Scale)	Time of Detection by the Local Strategy Using Light Microscopy (LM) in Comparison with the Time of Detection by Quantitative Real-Time Polymerase Chain Reaction (qPCR)		
	Same Visit	Following Visits (10–30 days Later)	Total
	*n* (%)	*n* (%)	*N* (%)
*P. vivax*			
<1/2+	24 (36.9)	16 (50.0)	40 (41.2)
1/2+	13 (20.0)	5 (15.6)	18 (18.6)
≥1+	28 (43.1)	11 (34.4)	39 (40.2)
Total	65 (100)	32 (100)	97 (100)
*P. falciparum*			
<1/2+	0 (0)	0 (0)	0 (0)
1/2+	0 (0)	0 (0)	0 (0)
≥1+	2 (100)	1 (100)	3 (100)
Total	2 (100)	1 (100)	3 (100)
Co-infection *			
<1/2+	0 (0)	1 (100)	1 (100)
1/2+	0 (0)	0 (0)	0 (0)
≥1+	0 (0)	0 (0)	0 (0)
Total	0 (0)	1 (100)	1 (100)

* P. vivax-P. falciparum.

**Table 5:** Evolution of clinical status of malaria infections detected by quantitative real-time polymerase chain reaction (qPCR).

Time of Detection of Malaria Infections by qPCR		All qPCR-Positive Malaria Infections					
		Symptomatic				Asymptomatic	Total
		In the Same Visit	In Following Visit (10-days Later)	After 2 Visits (20-days Later)	After 3 Visits (30-days Later)		
		*n* (%)	*n* (%)	*n* (%)	*n* (%)	*n* (%)	*N*
**Overall**							
	1st visit	76 (42.5)	18 (10.1)	10 (5.6)	2 (1.1)	73 (40.8)	179
	2nd visit	20 (19.8)	12 (11.9)	12 (11.9)	ND	57 (56.4)	101
	3rd visit	22 (23.7)	11 (11.8)	ND	ND	60 (64.5)	93
	4th visit	10 (13.3)	ND	ND	ND	65 (86.7)	75
	Total	128 (28.6)	41 (9.2)	22 (4.9)	2 (0.4)	255 (56.9)	448
***P. vivax***							
	1st visit	60 (44.1)	13 (9.6)	8 (5.9)	1 (0.7)	54 (39.7)	136
	2nd visit	18 (23.1)	10 (12.8)	8 (10.3)	ND	42 (53.8)	78
	3rd visit	22 (26.5)	10 (12.0)	ND	ND	51 (61.4)	83
	4th visit	8 (13.1)	ND	ND	ND	53 (86.9)	61
	Total	108 (30.2)	33 (9.2)	16 (4.5)	1 (0.3)	200 (55.9)	358
***P. falciparum***							
	1st visit	12 (41.4)	1 (3.4)	0 (0)	1 (3.4)	15 (51.7)	29
	2nd visit	2 (11.8)	0 (0)	4 (23.5)	ND	11 (64.7)	17
	3rd visit	0 (0)	1 (12.5)	ND	ND	7 (87.5)	8
	4th visit	2 (14.3)	ND	ND	ND	12 (85.7)	14
	Total	16 (23.5)	2 (2.9)	4 (5.9)	1 (1.5)	45 (66.2)	68
Co-infection *							
	1st visit	4 (28.6)	4 (28.6)	2 (14.3)	0 (0)	4 (28.6)	14
	2nd visit	0 (0)	2 (33.3)	0 (0)	ND	4 (66.7)	6
	3rd visit	0 (0)	0 (0)	ND	ND	2 (100)	2
	4th visit	0 (0)	ND	ND	ND	0 (0)	0
	Total	4 (18.2)	6 (27.3)	2 (9.1)	0 (0)	10 (45.5)	22

* P. vivax-P. falciparum. ND: no data.

**Table 6:** Univariate and multivariate risk factor analysis by malaria species.

Characteristics		%	*P. vivax*					*P. falciparum*					
			*n/N*	Univariate		Multivariate		%	*n/N*	Univariate		Multivariate	
				OR	[95% CI]	AOR	[95% CI]			OR	[95% CI]	AOR	[95% CI]
Gender													
	Female	46.8	183/391	Ref				9.21	36/391	Ref			
	Male	45.0	194/431	0.9	[0.7; 1.2]			12.8	55/431	1.5	[0.9; 2.4]	1.5	[0.9; 2.3] °
Age (years)													
	<5	37.0	54/146	Ref		Ref		7.53	11/146	Ref			
	5–14	50.0	121/242	1.7	[1.1; 2.6] *	1.7	[1.1; 2.6] *	11.6	28/242	1.8	[0.8; 3.9]	1.7	[0.8; 3.7]
	15–39	47.8	118/247	1.6	[1.0; 2.4] *	1.2	[0.7; 2.0]	10.9	27/247	1.6	[0.7; 3.5]	1.7	[0.7; 3.9]
	≥40	44.9	84/187	1.4	[0.9; 2.1]	0.9	[0.5; 1.7]	13.4	25/187	2.1	[1.0; 4.7] °	2.2	[0.9; 5.4] °
Main occupation													
	None or others	43.86	225/513	Ref		Ref		10.5	54/513	Ref			
	Farmer	49.2	152/309	1.3	[1.0; 1.7] °	1.6	[1.0; 2.4] *	12.0	37/309	1.2	[0.8; 1.9]	0.8	[0.4; 1.6]
Malaria episodes (previous 12 months)													
	0	45.7	289/633	Ref				11.1	70/633	Ref			
	≥1	46.5	87/187	1.1	[0.8; 1.5]			11.23	21/187	1.0	[0.6; 1.7]		
Travel (previos month)													
	No	46.3	341/736	Ref				11.1	82/736	Ref			
	Yes	41.9	36/86	0.9	[0.6; 1.4]			10.5	9/86	0.9	[0.4; 2.1]		
Housing structure (number of walls)													
	0	47.3	53/112	Ref				10.7	12/112	Ref			
	1–3	46.3	244/527	1.0	[0.6; 1.5]			10.4	55/527	1.0	[0.4; 2.3]		
	4	44.4	79/178	0.9	[0.5; 1.4]			12.9	23/178	1.4	[0.5; 3.5]		
Indoor residual spraying (previous 12 months)													
	No	35.9	19/53	Ref				13.21	7/53	Ref			
	Yes	46.5	353/759	1.4	[0.7; 2.5]			10.9	83/759	0.8	[0.3; 2.3]		
Electricity available													
	No	44.3	183/413	Ref				11.1	46/413	Ref			
	Yes	47.8	193/404	1.0	[0.7; 1.4]			10.9	44/404	0.9	[0.5; 1.6]		

* *p* < 0.05; ° *p* < 0.1; AOR: adjusted odds ratios; OR: crude odds ratios.

## Supplementary Materials

The following are available online at www.mdpi.com/1660-4601/15/12/2670/s1, Table S1: Evolution of clinical status of malaria infections detected and undetected by ACD-based strategy using light *microscopy* (LM).
