# Supplementary material for: Effectiveness of a Malaria Surveillance Strategy Based on Active Case Detection during High Transmission Season in the Peruvian Amazon
Source: Int J Environ Res Public Health. 2018 Nov 27;15(12):2670. doi: 10.3390/ijerph15122670 (PMC6314008; doi:10.3390/ijerph15122670)
Supplement: Supplementary file 1 [file ijerph-15-02670-s001.pdf]

Effectiveness of A Malaria Surveillance Strategy Based on Active Case Detection during High Transmission Season in the Peruvian Amazon

Diamantina Moreno-Gutierrez [1,2,3,*], Alejandro Llanos-Cuentas [4,5], José Luis Barboza [4], Juan Contreras-Mancilla [4,6], Dionicia Gamboa [4,6,7], Hugo Rodriguez [1], Gabriel Carrasco-Escobar [6,8], Raphaël Boreux [9], Marie-Pierre Hayette [9], Philippe Beutels [3], Niko Speybroeck [2,†] and Angel Rosas-Aguirre [2,4,†]

1 Facultad de Medicina Humana, Universidad Nacional de la Amazonía Peruana, Loreto 160, Peru; hmrodriguezf@hotmail.com (H.R.)
2 Research Institute of Health and Society (IRSS), Université catholique de Louvain, 1200 Brussels, Belgium; niko.speybroeck@uclouvain.be (N.S.); angelrosasa@gmail.com (A.R.-A.)
3 Centre for Health Economics Research and Modelling Infectious Diseases, Vaccine and Infectious Disease Institute, University of Antwerp, 2000 Antwerp, Belgium; philippe.beutels@uantwerpen.be
4 Instituto de Medicina Tropical "Alexander von Humboldt", Universidad Peruana Cayetano Heredia, Lima 31, Peru; alejandro.llanos.c@upch.pe (A.L.-C.); biobarbox@gmail.com (J.L.B.); juan.contreras.m@upch.pe (J.C.-M.); dionigamboa@yahoo.com (D.G.)
5 Facultad de Salud Pública y Administración, Universidad Peruana Cayetano Heredia, Lima 31, Peru.
6 Laboratorios de Investigación y Desarrollo, Facultad de Ciencias y Filosofia, Universidad Peruana Cayetano Heredia, Lima 31, Peru; gabriel.carrasco@upch.pe (G.C.-E)
7 Departamento de Ciencias Celulares y Moleculares, Facultad de Ciencias y Filosofia, Universidad Peruana Cayetano Heredia, Lima 31, Peru
8 Division of Infectious Diseases, Department of Medicine, University of California San Diego, La Jolla, CA 92093, USA
9 Department of Clinical Microbiology, Center for Interdisciplinary Research on Medicines (CIRM), University Hospital of Liège, 4000 Liège, Belgium; raphael.boreux@chuliege.be (R.B.); mphayette@chuliege.be (M.-P.H.)
* Correspondence: dlmorenog@gmail.com
† These authors contributed equally to this work.

SUPPLEMENTARY MATERIAL

Table S1. Evolution of clinical status of malaria infections detected and undetected by ACD-based strategy using light *microscopy* (LM)

Time of detection of malaria infections by qPCR	qPCR-positive malaria infections detected by ACD-based strategy using *light microscopy* (LM)						qPCR-positive malaria infections undetected by ACD-based strategy using *light microscopy* (LM)					
	Symptomatic				Asymptomatic	Total	Symptomatic				Asymptomatic	Total
	In the same visit	In following visit (10-d later)	After 2 visits (20-d later)	After 3 visits (30-d later)			In the same visit	In following visit (10-d later)	After 2 visits (20-d later)	After 3 visits (30-d later)		
	n (%)	n (%)	n (%)	n (%)	n (%)	N	n (%)	n (%)	n (%)	n (%)	n (%)	N
**Overall**												
1st visit	20 (47.6)	5 (11.9)	2 (4.8)	0 (0)	15 (35.7)	42	56 (40.9)	13 (9.5)	8 (5.8)	2 (1.5)	58 (42.3)	137
2nd visit	5 (25.0)	4 (20.0)	2 (10.0)	ND	9 (45.0)	20	15 (18.5)	8 (9.9)	10 (12.3)	ND	48 (59.3)	81
3rd visit	14 (53.8)	3 (11.5)	ND	ND	9 (34.6)	26	8 (11.9)	8 (11.9)	ND	ND	51 (76.1)	67
4th visit	2 (15.4)	ND	ND	ND	11 (84.6)	13	8 (12.9)	ND	ND	ND	54 (87.1)	62
Total	41 (40.6)	12 (11.9)	4 (4.0)	0 (0)	44 (43.6)	101	87 (25.1)	29 (8.4)	18 (5.2)	2 (0.6)	211 (60.8)	347
P. vivax												
1st visit	17 (44.7)	4 (10.5)	2 (5.3)	0 (0)	15 (39.5)	38	43 (43.9)	9 (9.2)	6 (6.1)	1 (1.0)	39 (39.8)	98
2nd visit	5 (15.6)	4 (12.5)	2 (6.3)	ND	21 (65.6)	32	13 (28.3)	6 (13.0)	6 (13.0)	ND	21 (45.7)	46
3rd visit	14 (42.4)	3 (9.1)	ND	ND	16 (48.5)	33	8 (16.0)	7 (14.0)	ND	ND	35 (70.0)	50
4th visit	2 (15.4)	ND	ND	ND	11 (84.6)	13	6 (12.5)	ND	ND	ND	42 (87.5)	48
Total	38 (39.2)	11 (11.3)	4 (4.1)	0 (0)	44 (45.4)	97	70 (26.8)	22 (8.4)	12 (4.6)	1 (0.4)	156 (59.8)	261
P. falciparum												
1st visit	3 (100)	0 (0)	0 (0)	0 (0)	0 (0)	3	9 (34.6)	1 (3.8)	0 (0)	1 (3.8)	15 (57.7)	26
2nd visit	0 (0)	0 (0)	0 (0)	ND	0 (0)	0	2 (11.8)	0 (0)	4 (23.5)	ND	11 (64.7)	17
3rd visit	0 (0)	0 (0)	ND	ND	0 (0)	0	0 (0)	1 (12.5)	ND	ND	7 (87.5)	8
4th visit	0 (0)	ND	ND	ND	0 (0)	0	2 (14.3)	ND	ND	ND	12 (85.7)	14
Total	3 (100)	0 (0)	0 (0)	0 (0)	0 (0)	3	13 (20.0)	2 (3.1)	4 (6.2)	1 (1.5)	45 (69.2)	65
**Co-infection***												
1st visit	0 (0)	1 (100)	0 (0)	0 (0)	0 (0)	1	4 (30.8)	3 (23.1)	2 (15.4)	0 (0)	4 (30.8)	13
2nd visit	0 (0)	0 (0)	0 (0)	ND	0 (0)	0	0 (0)	2 (33.3)	0 (0)	ND	4 (66.7)	6
3rd visit	0 (0)	0 (0)	ND	ND	0 (0)	0	0 (0)	0 (0)	ND	ND	2 (100)	2
4th visit	0 (0)	ND	ND	ND	0 (0)	0	0 (0)	ND	ND	ND	0 (0)	0
Total	0 (0)	1 (100)	0 (0)	0 (0)	0 (0)	1	4 (19.0)	5 (23.8)	2 (9.5)	0 (0)	10 (47.6)	21

**P. vivax-P. falciparum*. ND: no data
